# Gas Barrier Properties of Organoclay-Reinforced Polyamide 6 Nanocomposite Liners for Type IV Hydrogen Storage Vessels

**DOI:** 10.3390/nano15141101

**Published:** 2025-07-16

**Authors:** Dávid István Kis, Pál Hansághy, Attila Bata, Nándor Nemestóthy, Péter Gerse, Ferenc Tajti, Eszter Kókai

**Affiliations:** 1 Department of Automotive Technologies, Faculty of Transportation Engineering and Vehicle Engineering, Budapest University of Technology and Economics, Műegyetem rkp. 3, H-1111 Budapest, Hungary; 2 Department of Innovative Vehicles and Materials, GAMF Faculty of Mechanical Engineering and Computer Science, John von Neumann University, Izsáki út 10, H-6000 Kecskemét, Hungary; hansaghy.pal@nje.hu (P.H.); bata.attila@nje.hu (A.B.); gerse.peter@nje.hu (P.G.); tajti.ferenc@nje.hu (F.T.); 3 Research Group on Bioengineering, Membrane Technology and Energetics, University of Pannonia, Egyetem u. 10, H-8200 Veszprém, Hungary; nemestothy.nandor@mk.uni-pannon.hu; 4 Department of Applied Sustainability, Széchenyi István University, Egyetem tér 1, H-9026 Győr, Hungary

**Keywords:** hydrogen permeability, polyamide 6 nanocomposite, organoclay, pressure vessel, crystallinity

## Abstract

This study investigates the hydrogen permeability of injection-molded polyamide 6 (PA6) nanocomposites reinforced with organo-modified montmorillonite (OMMT) at varying concentrations (1, 2.5, 5, and 10 wt. %) for potential use as Type IV composite-overwrapped pressure vessel (COPV) liners. While previous work examined their mechanical properties, this study focuses on their crystallinity, morphology, and gas barrier performance. The precise inorganic content was determined using thermal gravimetry analysis (TGA), while differential scanning calorimetry (DSC), wide-angle X-ray diffraction (WAXD), and scanning electron microscopy (SEM) were used to characterize the structural and morphological changes induced by varying filler content. The results showed that generally higher OMMT concentrations promoted γ-phase formation but also led to increased agglomeration and reduced crystallinity. The PA6/OMMT-1 wt. % sample stood out with higher crystallinity, well-dispersed clay, and low hydrogen permeability. In contrast, the PA6/OMMT-2.5 and -5 wt. % samples showed increased permeability, which corresponded to WAXD and SEM evidence of agglomeration and DSC results indicating a lower degree of crystallinity. PA6/OMMT-10 wt. % showed the most-reduced hydrogen permeability compared to all other samples. This improvement, however, is attributed to a tortuous path effect created by the high filler loading rather than optimal crystallinity or dispersion. SEM images revealed significant OMMT agglomeration, and DSC analysis confirmed reduced crystallinity, indicating that despite the excellent barrier performance, the compromised microstructure may negatively impact mechanical reliability, showing PA6/OMMT-1 wt. % to be the most balanced candidate combining both mechanical integrity and hydrogen impermeability for Type IV COPV liners.

## 1. Introduction

Type IV composite-overwrapped pressure vessels, commonly used in hydrogen-powered vehicles, consist of a polymer liner completely encased in a fiber-reinforced composite structure. The liner provides hydrogen containment, while the composite shell withstands the internal pressure [1]. There is increased interest in the processing of polymer nanocomposites instead of neat polymers as liner materials for COPVs. The gas permeability property of polymer-layered silicate nanocomposites is an important consideration for hydrogen storage purposes. However, it is crucial to understand the thermal properties of the nanocomposites, which provide information relevant to the manufacturing technology of hydrogen tanks. These studies are not only essential in the research of the materials used in hydrogen storage but also expand the knowledge about polymer-based nanocomposites by determining the properties of the raw materials. The aim of this work is to investigate injection-molded PA6 nanocomposites with a wide range of organoclay contents (1; 2.5; 5; 10 wt. %) by means of TGA, DSC, WAXD, SEM, and permeability analysis to discuss the effect of organoclay content on the crystalline structure and hydrogen permeability. Polyamide is one of the most used polymer liners in hydrogen storage vessels [2]. OMMT is a layered silicate that can reduce the gas permeability of the material, thereby decreasing hydrogen diffusion [3]. The materials discussed have already been the subject of an extensive study with respect to mechanical properties, the results of which have been published elsewhere [4].

PA6 is a highly crystalline thermoplastic polymer, with polar groups along the molecular chains that naturally enhance their capacity to penetrate the interlayer spaces of inorganic nanofillers. Organoclay nanofillers are often treated with organic compounds to enhance organoclay dispersion within the polymer matrix and improve the adhesion between the polymer and organoclay. This typically leads to more favorable morphologies in PA6 nanocomposites, such as exfoliated or at least intercalated structures [5,6].

Crystallinity is often desired, as it provides strength, stiffness, and temperature stability. Semicrystalline polymer composites, such as PA6/OMMT nanocomposites, are a three-phase system, comprising layered silicate, amorphous polymer, and crystalline polymer regions. The fact that PA6 can exhibit polymorphism by melt processing with two stable crystalline phases—a monoclinic α-phase and a pseudo-hexagonal γ-phase—further confirms the need for the investigation of composites. PA6 primarily crystallizes in the α-phase, characterized by fully extended, planar zigzag polymer chains arranged into planar sheets bonded by hydrogen. This α-phase is thermodynamically stable and is typically formed through slow cooling and high crystallization temperatures [7]. In contrast, the γ-phase is the less thermodynamically stable form with a shorter chain axis due to the tilting of amide groups relative to the chain axis. In the γ-phase, hydrogen bonds form between parallel polymer chains, which requires the amide linkages to twist out of the plane. This results in a pseudo-hexagonal crystal structure.

Variation in the crystalline structure of PA6 and its nanocomposites has been reported by many different studies, which may be due to the large differences in the base material processing conditions. Bureau et al. claimed that crystalline forms in PA6/OMMT nanocomposites remained qualitatively the same for different compression-molding conditions [8]. Additionally, no significant levels of the γ-crystalline phase could be generated in a neat compression-molded PA6 specimen. However, Picard et al. evidenced both α- and γ-phases in neat PA6 samples produced by film-blowing [9]. The effect of the clay concentration on the crystalline structure and permeability of injection-molded PA6/OMMT nanocomposites—characterized by rapid cooling—remains largely unexplored in the literature.

In polymer nanocomposites, gas barrier properties are governed by the solution–diffusion mechanism, wherein permeant molecules dissolve into the polymer matrix and diffuse through it under a chemical potential gradient [10]. Both the diffusion and solubility of hydrogen are influenced by the amorphous and crystalline regions, as hydrogen predominantly permeates through the amorphous phase. Crystalline phases act as impermeable barriers, while nanofiller dispersion introduces a tortuous path that lengthens the diffusion path and reduces permeability. As confirmed in earlier studies [9,11], the degree of filler exfoliation, aspect ratio, and spatial orientation critically affect gas transport by physically obstructing diffusion and altering the available free volume. Furthermore, rigid filler structures reduce chain mobility, hindering molecular-scale interactions with hydrogen. In this context, it becomes important to understand how organoclay nanofillers influence the crystalline morphology of the polymer matrix, as this directly affects gas transport. Therefore, it is crucial to consider any crystalline changes in the polymer matrix in the presence of organoclay particles to understand the evolution of gas transport phenomena in COPVs. Injection molding is one possible way to produce COPV liners [12]. It has been shown that processing modes coupled with rapid cooling such as injection molding result in the coexistence of α- and γ-phases [9,13]. According to Anoukou et al., it has been well evidenced that the organoclay nanoparticles also induce crystal transformation from the α-phase to the γ-phase, and γ-lamellae are expected to grow on the OMMT sheets [14]. Specifically, their proposal included two morphological models for this transformation: in one, γ-crystallites form independently in the matrix, while in the second, they grow directly as an interphase layer coating the clay surfaces. The latter is supported by experimental data showing that OMMT platelets act as heterogeneous nucleation sites by locally lowering the activation energy barrier for crystallization, favoring the formation of γ-crystals over the thermodynamically more stable α-form. Yoon et al. confirmed that OMMT induces the heterogeneous nucleation of the γ-form, resulting in an increase in the crystallization rate [15]. Isothermal crystallization experiments on PA6/OMMT fibers showed that the overall crystallization rate increased with OMMT content. This is attributed to the ability of the clay surfaces to promote nucleation while restricting chain folding, thus facilitating γ-phase formation during rapid cooling and even resisting full transformation to the α-form during drawing. Varlot et al. concluded that OMMT actually prevents crystallization in the α-form and promotes the γ-form [16]. Furthermore, the relative fraction of α- and γ-crystalline structures is found to be dependent on the filler content and the interfacial interaction between PA6 and the silicate layers. Miltner et al. observed with DSC results that the relative amount of the γ-crystalline fraction with respect to the α-fraction strongly depends on the surfactant. Neat clay-based nanocomposites had a melting behavior close to that of unfilled PA6, since they contain essentially α-crystals, while organo-modified nanocomposites had significantly lower melting temperatures due to the presence of γ-crystals [13]. Varlot et al. studied OMMT nanofillers with different surfactant contents in PA6 matrix and found that an exfoliated nanocomposite was obtained with slightly swollen OMMT, while highly swollen OMMT resulted in intercalated systems [16]. There are also disagreements in the literature on whether the crystallinity of PA6 remains unchanged or decreases with the increased amount of OMMT. Picard et al. reported that the crystallinity did not change with the increased amount of OMMT nanofillers in PA6/OMMT films based on DSC data [9]. Wu et al., on the other hand, reported that neat PA6 has a higher crystallinity than PA6/OMMT under low cooling rates [6].

Although the current study concentrates on the gas barrier performance and crystalline morphology of PA6/OMMT nanocomposites, it is important to note that the liner’s interaction with the metallic boss is a critical consideration in practical tank design. As recent investigations have shown, treatments such as sandblasting and polyethylene grafting can significantly enhance the fatigue performance and adhesion strength at the metal–polymer interface [17,18]. Future studies may aim to correlate such mechanical interface optimization with the barrier and thermal behavior explored here.

In summary, the literature on polyamide 6 (PA6) nanocomposites, particularly with organo-modified montmorillonite (OMMT), highlights the critical role of nanofiller content and processing conditions in influencing the crystalline structure. While several studies have demonstrated the potential of OMMT to enhance the formation of the γ-crystalline phase and alter the α-phase, the findings remain variable depending on factors like cooling rates, polymerization techniques, and nanofiller dispersion. The target of this paper is to further explore the crystallinity of injection-molded PA6/OMMT nanocomposites, correlating the organoclay content with changes in the hydrogen gas barrier performance of Type IV COPV liners.

The remainder of this paper is organized as follows: Section 2 describes the materials and experimental methods used in the study. Section 3 presents and discusses the results of thermal, structural, and gas permeability analyses. Finally, Section 4 concludes the paper by summarizing the key findings and highlighting directions for future research.

## 2. Materials and Methods

This research forms part of a larger series of studies on the performance of PA6/OMMT nanocomposites for use in Type IV composite-overwrapped pressure vessel (COPV) liners. The study is segmented into two key parts: mechanical analysis and gas barrier properties. In the first part, as detailed in our previous publication, mechanical testing (including tensile tests and dynamic mechanical analysis) was carried out to assess the mechanical properties of PA6/OMMT nanocomposites with the same organoclay concentrations as in the present paper (1, 2.5, 5, and 10 wt. %) [4]. It was found that the PA6/OMMT-1 wt. % composition provided the best mechanical performance in terms of the yield strength and ductility. The dynamic mechanical analysis results showed the preservation of the amorphous–crystalline balance of neat PA6 in the case of the PA6/OMMT-1 and -2.5 wt. % composites. The current study builds upon these findings by focusing on structural aspects, specifically investigating how the OMMT content affects the crystalline morphology and hydrogen permeability of these nanocomposites.

### 2.1. Sample Preparation

Neat polyamide 6 powder, trademarked as Alphalon^®^ 24 (Grupa Azoty S.A., Tarnów, Poland), was used as the matrix material. The montmorillonite organoclay was Nanomer^®^ I.34TCN (Nanocor Inc., Hoffman Estates, IL, USA) with the organic surfactant methyl octadecyl bis-2-hydroxyethyl ammonium methylsulfate. The filler concentration of PA6/OMMT nanocomposites was chosen based on the literature data: 1, 2.5, 5, and 10 wt. % [19,20,21,22]. The composites were developed using an extrusion–injection process: (1) Weigh an appropriate amount of OMMT. (2) Shake the composite powder in a sealed bucket to create a uniform mixture. (3) Melt-compound the neat and composite materials with an LHFS1-271022-type twin-screw extruder (Labtech Ltd., Hopkinton, MA, USA). (4) Granulate the extruded filaments to a uniform size. The parameters of the extrusion and injection molding are shown in Table 1 and Table 2, respectively. The test specimens from the previous research and the plate-shaped specimens for the current study were both made from granules produced in the same manufacturing batch. Both the neat PA6 and PA6/OMMT nanocomposites were dried at 80 °C for 6 h. Then, the fully dried material was used to injection-mold rectangular sheets on an e-mac 80-type injection-molding machine (Engel GmbH., Schwertberg, Austria) with a length, width, and thickness of 97 mm, 50 mm, and 2.5 mm, respectively. All specimens were conditioned for 1 week prior to testing (23 °C ± 2 °C, 50% ± 10% humidity).

### 2.2. Characterization Methods

TGA was used to determine the precise weight fraction of the inorganic content. The experiments were performed on granules with a TGA Q5000-type (TA Instruments Inc., New Castle, DE, USA) Thermogravimetric Analyzer from 40 to 800 °C at 10 °C/min under a nitrogen atmosphere.

The crystallinity of the samples was determined using a DSC Q200-type (TA Instruments Inc., New Castle, DE, USA) differential scanning calorimeter which measures the heat flow associated with thermal transitions such as melting and crystallization. Experiments were carried out in a nitrogen atmosphere. All specimens were in the range of 4–5 mg, cut from the injection-molded specimen. The melting temperature was obtained by the following process: (1) The samples were heated from 30 °C to 280 °C at a rate of 10 °C/min, holding for 5 min to remove the thermal history. (2) The samples were then cooled to 120 °C at a rate of 1, 5, 10, 20, 30, or 50 °C/min, held for 1 min, and heated to 280 °C at a rate of 10 °C/min and held for 1 min, and the cycle repeated with another cooling rate.

The crystallinity index was calculated for each nanocomposite using the following equation [15]:

(1) $$X(\mathrm{per}\;\mathrm{cent})=\frac{{∆H}_{f}}{\left(1-\phi \right)\Delta H_{m}^{0}}\times 100$$

where ${∆H}_{f}$ is the heat of fusion, $\Delta H_{m}^{0}$ represents the extrapolated enthalpy value for the melting of a sample with 100% crystallinity taking a value of 190 J/g [9,15,23], and ϕ is the weight fraction of the filler.

Clay dispersion inside the injection-molded sheets was analyzed using X-ray diffraction, a technique used to determine the crystalline structure and interlayer spacing in polymer nanocomposites, for example. All nanocomposite samples were illuminated with a MiniFlex II (Rigaku, Tokyo, Japan) instrument with a CuKα target (λ = 1.541 Å) at room temperature in the range of 2° to 90°. The X-ray generator was operated at 30 kV with a scan speed of 2°/min. The planes responsible for the diffraction pattern are oriented parallel to the surface of the sample. The interlayer spacing (d_hkl_) was calculated from the (001) peak by using Bragg’s law.

The nanoparticle structure was examined using scanning electron microscopy (SEM), model Sigma 300 VP (Carl Zeiss, Oberkochen, Germany). Samples were prepared for SEM observations by breaking injection-molded specimens in liquid nitrogen. Following the method of Liu et al., fracture surfaces were etched by tetrahydrofuran for 24 h prior to investigation [24].

Sheets were assembled into a CF042 (Sterlitech, Kent, WA, USA) permeation cell consisting of two compartments separated by the specimen (effective area: 35 cm^2^). The cell temperature was maintained at 20 ± 1 °C. The system was first evacuated to remove all traces of air, and then the upper compartment was flushed with hydrogen, allowing the sample to equilibrate. The pressure of the upstream compartment was set to 2 MPa (20 bar). The permeability coefficient was calculated from the slope of the steady-state line and expressed in mol/m·s·Pa. Since all samples had the same thickness, the hydrogen permeability could be directly correlated with the nanocomposite composition.

This study was designed as a comparative investigation to evaluate material trends across different nanocomposites. Although statistical repetitions were not included, all tests were conducted under consistent conditions, and standardized preparation protocols were followed to ensure the internal comparability of the results.

## 3. Results and Discussion

As both silicate layers and crystalline lamellae are considered to be impermeable to hydrogen molecules, it is important to describe precisely the amount and dispersion of montmorillonite in the PA6 matrix to explain the gas transport properties.

### 3.1. Actual Clay Content

**Figure 1:**
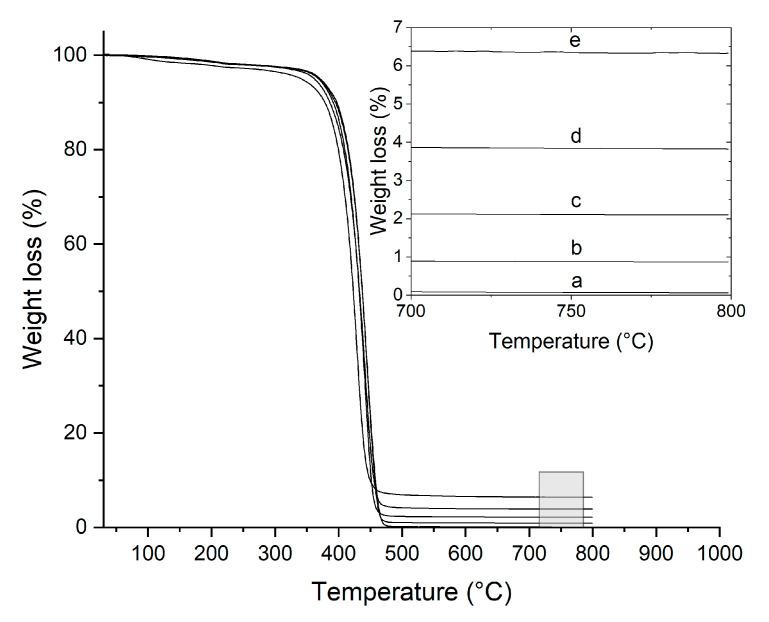
Weight loss curves determined by TGA at a heating rate of 10 °C/min for (**a**) neat PA6 and its nanocomposites: (**b**) PA6/OMMT-1; (**c**) PA6/OMMT-2.5; (**d**) PA6/OMMT-5; and (**e**) PA6/OMMT-10.

The method of mixing powdered organoclay with polymer powder before melt compounding is ideal for achieving a homogeneous composition. However, deviations from intended compositions may still occur. Thus, TGA analysis was conducted on the nanocomposite granules to confirm the actual inorganic content in the composites. The results yielded an organoclay content of 1.25, 2.99, 5.46, and 9.04 wt. % in the PA6/OMMT-1, -2.5, -5, and -10 samples, respectively. The reported loading values correspond to the organoclay, as it was the component originally weighed. The actual silicate content, which is plotted in Figure 1, is 30% lower than these values.

Due to deviations of up to ~30% between the nominal and measured clay content, all plots in this study are based on the TGA-determined actual filler content rather than nominal composition.

### 3.2. Crystalline Structure

**Figure 2:**
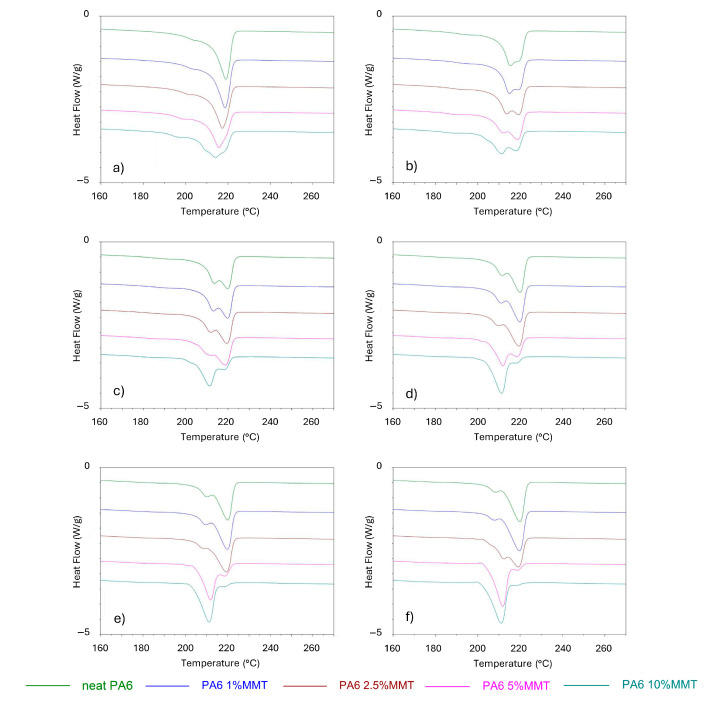
DSC heating scans after cooling cycles with different rates of (**a**) 1 °C/min; (**b**) 5 °C/min; (**c**) 10 °C/min; (**d**) 20 °C/min; (**e**) 30 °C/min; and (**f**) 50 °C/min. Exotherm peaks are upwards. The curves are vertically offset for clarity.

To obtain more information about the polymorphic behavior of PA6/OMMT composites, DSC thermograms were used. Figure 2 shows information about the structural change from heating scans after cooling at different rates in a range of between 1 and 50 °C/min. Figure 2a shows the heating scans of PA6 and PA6/OMMT nanocomposites after cooling at the slowest cooling rate (1 °C/min), where all curves contain one dominant melting peak, corresponding to the higher-temperature α-form, except for PA6/OMMT-10 wt. % which has a broad peak suggesting a mixture of the two crystalline forms. All heating scans from 5 °C/min have two melting peaks. Based on Picard et al., it can be concluded that the high-temperature peak corresponds to the α-form and the low-temperature peak corresponds to the γ-form [9]. At 5 °C/min (Figure 2b), the crystalline fraction is almost identical for compositions up to 5 wt. %, and the shape of the peaks suggests an equal amount of α- and γ-formation.

From 10 °C/min (Figure 2c), the γ-crystal fraction is observed to increase at the expense of the α-crystal fraction, which is in agreement with the results of Wu et al., who found similar trends at the same cooling rates [6]. Generally, the nature of low-filler-content composites (PA6/OMMT-1 and -2.5 wt. %) is almost identical to neat PA6 at all cooling rates and is mainly dominated by the α-phase. The crystallization of the γ-phase first predominates in the PA6/OMMT-10 wt. % sample at a 10 °C/min cooling rate (Figure 2c), and its fraction keeps growing with a higher cooling rate. At higher cooling rates, the molecular mobility decreases, promoting γ-phase formation, which is kinetically favored over the α-phase in the fast cooling process. Such behavior suggests that the organoclay filler serves as a nucleating agent, facilitating γ-phase crystallization. As depicted in Figure 2d, at a 20 °C/min cooling rate, γ-phase crystallization prevails in the PA6/OMMT-5 wt. % sample, as well. In the case of the PA6/OMMT-5 and PA6/OMMT-10 wt. % specimens, the highest 50 °C/min cooling rate (Figure 2f) presents only one melting peak, corresponding to the lower-temperature γ-form, and only a trace of the α-form peak. The increasing fraction of γ-phase with organoclay content is logical due to the heterogeneous crystallization role of the filler in γ-formations.

These observations indicate that the filler content significantly influences the phase behavior of PA6. For instance, the PA6/OMMT-5 and PA6/OMMT-10 wt. % samples exhibit a shift from the α-phase to the γ-phase with increased cooling rates, highlighting the role of the filler in accelerating γ-phase nucleation. This trend contrasts with the low-filler-content samples, where the α-phase remains predominant even at higher cooling rates. Such findings point to the ability of organoclay to act as a heterogeneous nucleation site, particularly favoring γ-crystal formation in high-filler-content samples. This behavior can be explained by the local reduction in free energy barriers at the OMMT–polymer interface, where the surface of the clay promotes γ-phase lamellae formation. The proximity of PA6 chains to the silicate layers induces chain alignment and enhances nucleation rates during rapid cooling. Moreover, this localized crystallization influences hydrogen transport by modifying the density and orientation of impermeable crystalline domains. Reduced chain mobility near the interface further limits hydrogen solubility. These coupled effects establish a clear mechanism by which OMMT not only alters crystallinity but also governs gas barrier behavior at the molecular level.

**Figure 3:**
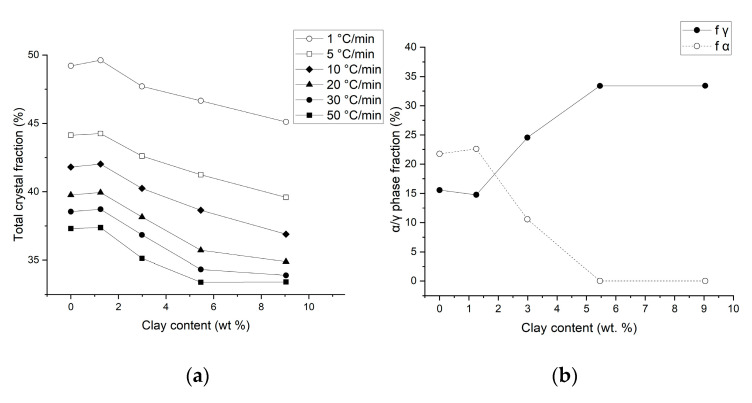
Plots of the (**a**) total crystallinity as a function of the cooling rate before the heating cycle and (**b**) fraction of α- and γ-crystalline phases calculated from heating scans after 50 °C/min cooling as a function of the clay content.

The total crystallinity based on the heat of fusion is calculated according to Equation (1) and is confirmed to generally decrease with the increase in the cooling rate, as depicted in Figure 3a. The exception was the PA6/OMMT-1 wt. % sample, which showed the highest level of crystallinity compared to the other nanocomposites and the neat PA6, independent of cooling rates. This is a new observation compared to some literature data [9,15]. This unique observation for PA6/OMMT-1 wt. % suggests that a lower filler content promotes higher crystallinity, potentially due to the improved dispersion of smaller organoclay particles, which limits the disruption of polymer chain mobility. This finding contradicts some previous reports, which typically show reduced crystallinity with increased filler content. The balance between the filler and cooling rate in these low-filler systems may be optimized to enhance the overall crystallinity, providing new insights into processing these nanocomposites.

To quantify the relative fractions of the α- and γ-crystalline forms, peak deconvolution of the DSC heating curves was performed following 50 °C/min cooling, which best replicates injection-molding conditions. The overlapping melting peaks were fitted using Gaussian functions, and the integrated areas corresponding to each crystalline form were used to determine their individual contributions to the total crystallinity. Figure 3b provides a quantitative confirmation of the qualitative observations shown in Figure 2f. The γ-phase contributed 41.73% of the total melting enthalpy in neat PA6 and 39.5 in the PA6/OMMT-1 wt. % sample, while this value was 69.94% in the PA6/OMMT-2.5 wt. % sample and 100% at higher concentrations.

Figure 3b also provides an explanation to our previous study [4], which investigated the same PA6/OMMT nanocomposites and revealed a linear increase in the Young’s modulus with increasing OMMT content. This trend correlates well with the rise in the γ-phase content observed in the current work. The quantitative relationship between crystallinity and mechanical properties has been extensively investigated across various polymer nanocomposite systems. Masenelli-Varlot et al. [25] also reported a nearly linear increase in the Young’s modulus of PA6/clay nanocomposites as a function of filler content, which coincided with changes in the crystalline morphology. They revealed γ-lamellae growing from exfoliated clay surfaces, supporting efficient stress transfer between the matrix and filler via crystalline reinforcement. Another similarity can be observed when comparing the tensile yield strength from the previous paper [4] and crystallinity data from the present one: only the PA6/OMMT-1 wt. % sample showed a higher yield strength than the neat PA6—for example, an increase of 11% at 85 °C—which mirrors the trend of total crystallinity.

It is important to mention that Lincoln et al. reached a different conclusion with DSC measurements and the X-ray characterization of PA6/OMMT nanocomposites with 2 and 5 wt. % OMMT content [26]. The DSC results reflected a general decrease in crystallinity with an increased fraction of OMMT in compression-molded PA6/OMMT samples. At the same time, data from wide-angle X-ray scattering showed that the effect of the OMMT concentration was different from that inferred from DSC measurements. Not only was the crystallinity higher at higher OMMT concentrations but the significance of the α-phase prior to melting was also scaled to be lower than what followed from the endotherms of DSC curves. This discrepancy underscores the need for complementary techniques like X-ray diffraction to fully characterize the crystalline structure and gain a comprehensive understanding of the filler’s impact on crystallinity.

### 3.3. Dispersion State of Nanofillers

**Figure 4:**
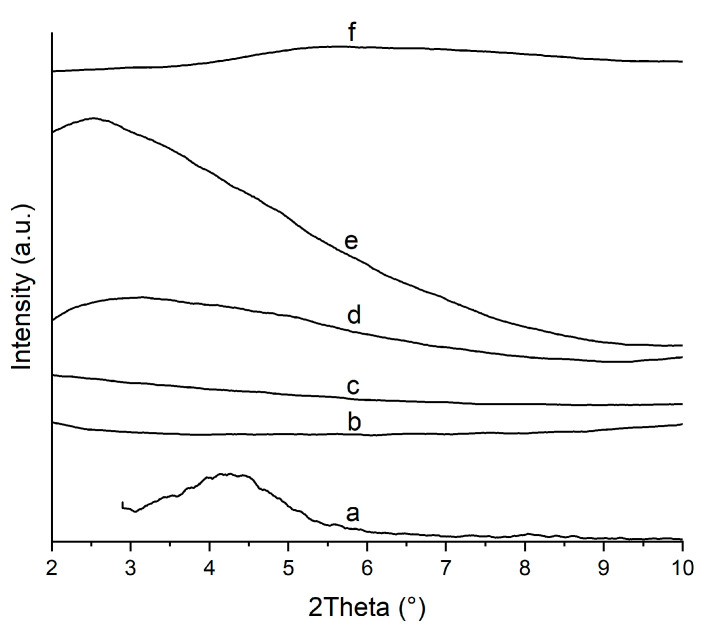
WAXD patterns for (**a**) pristine OMMT powder, (**b**) neat PA6 matrix, and nanocomposites (**c**) PA6/OMMT-1, (**d**) PA6/OMMT-2.5, (**e**) PA6/OMMT-5, and (**f**) PA6/OMMT-10 in a small-angle range (shifted curves).

Figure 4 shows the WAXD patterns of pristine OMMT, neat PA6, and PA6/OMMT nanocomposites for the 2Theta range from 2° to 10°. X-ray diffraction data below 10° can be used to determine whether the clay platelets in the nanocomposites were exfoliated or intercalated within the polymer matrix. A strong peak at 2Theta = 4.3° displayed by the montmorillonite corresponds to the mean interlayer spacing in the silicate of the (001) plane to d_(001)_ = 20.5 Å, according to Bragg’s law. This is in very good agreement with the technical data sheet (18–22 Å). The PA6 matrix pattern is shown as a reference for comparing the diffraction peaks originating from the dispersed clay layers in the PA6 matrix. The absence of the small-angle peak in the PA6/OMMT-1 wt. % sample indicates that the silicate layers of OMMT were exfoliated and dispersed in the PA6 matrix, explained by the fact that single sheets cannot give rise to a diffraction pattern [6,20]. Clear diffraction peaks are observed for nanocomposites with a higher filler content, indicating intercalation. Looking at the peaks of PA6/OMMT-2.5 and PA6/OMMT-5 wt. %, the shift of the small-angle diffraction peaks to smaller angles can be observed, meaning that the basal spacing increases with the increasing clay content, as shown in Table 3. The PA6/OMMT-2.5 and PA6/OMMT-5 wt. % samples show a broad diffraction peak at 2Theta = 3.3° and 2Theta = 2.5°, respectively, indicating a disordered intercalated structure, similar to what was observed by Sinha Ray et al. on PBS/saponite nanocomposites with 5.5 wt. % organoclay loading [27]. The PA6/OMMT-10 wt. % system showed only a low-intensity peak, indicating a lack of order.

**Figure 5:**
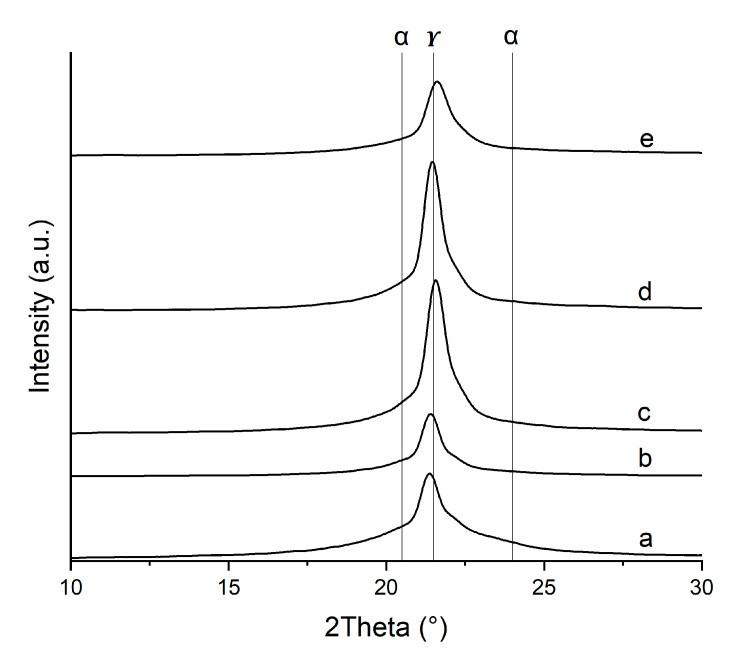
WAXD scans of (**a**) neat PA6 matrix and nanocomposites, (**b**) PA6/OMMT-1, (**c**) PA6/OMMT-2.5, (**d**) PA6/OMMT-5, and (**e**) PA6/OMMT-10, in a high-angle range (shifted curves).

Figure 5 presents the WAXD patterns of PA6/OMMT nanocomposites in the 2Theta range from 15° to 30°. One intense peak, located around 2Theta = 21.5°, was observed for all compositions, which is characteristic of the (200) and (101) planes of the γ-phase [9]. Neat PA6 shows a broad peak, suggesting a mixture of the two crystalline forms. The location of shoulder peaks at 2Theta = 20° and 24° can be assigned to the (200) and (002) planes of the α-crystal formation, respectively [16]. The minor contribution of the α-form crystalline peaks agrees with our DSC results and can be explained with the rapid cooling conditions during injection molding. Varlot et al. reported a similar crystalline structure in injection-molded PA6/OMMT nanocomposite samples [16]. Adame also reported the higher dominance of γ-form crystals over α-forms with increasing OMMT content in PA6/OMMT samples processed by film extrusion [20].

The diffraction pattern of PA6/OMMT-1 closely resembles that of the neat PA6, suggesting that the filler is well dispersed without significantly altering the phase composition. The presence of the gamma phase remains unchanged by the filler. This trend is further supported by DSC thermograms, which show a strong similarity between the neat polymer and the 1 wt. % composite. In the case of PA6/OMMT-2.5, the gamma phase dominates, but traces of the alpha phase are still detectable, as indicated by the shouldered WAXD pattern. Shoulder peaks are not observable for higher-clay-content compositions.

Although the wide-angle X-ray diffraction (WAXD) patterns confirmed the presence of crystalline α- and γ-phases, the quantitative analysis of relative crystallinity from the WAXD data was not performed. Instead, differential scanning calorimetry (DSC) was used as the primary method for crystallinity determination due to its higher sensitivity to phase transitions in PA6-based systems.

**Figure 6:**
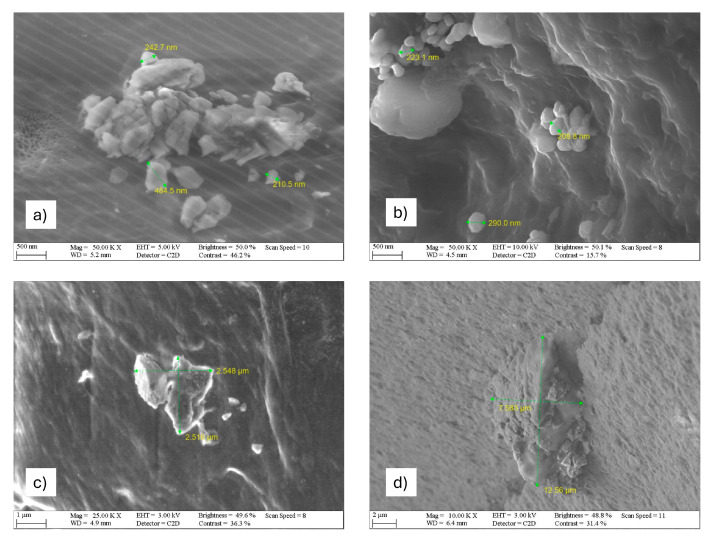
SEM images of (**a**) PA6/OMMT-1%; (**b**) PA6/OMMT-2.5%; (**c**) PA6/OMMT-5%; and (**d**) PA6/OMMT-10%.

The interface model representation provided by Anoukou et al. claims that the γ-crystal phase forms an interphase coating around the organoclay particles, suggesting the heterogeneous nucleation of γ-form crystals near the organoclay surface [14]. These coated particles are embedded in the amorphous part of PA6 considered as the matrix, while α-phase crystallites are located further away. Yoon et al. explains that the α-phase can only form at a distance from the silicate surface where the ability of the chains to fold is not interrupted [15]. Images supporting the morphology outlined in the literature were obtained with SEM, as shown in Figure 6. Picard et al. provided a quantitative description of the montmorillonite particle dispersion [9], which considered the polydispersity of the filler sizes from individual exfoliated sheets to micron-sized agglomerates. In the case of low-filler-content composites (PA6/OMMT-1 and PA6/OMMT-2.5 wt. %, Figure 6a,b, respectively), the particle sizes are in the range of a few hundred nanometers, which was predicted by the models of Picard et al. in connection with middle- and micron-sized agglomerates. However, the particles in the PA6/OMMT-5 and PA6/OMMT-10 wt. % (Figure 6c,d, respectively) specimens appear as irregularly shaped, few-thousand-nanometer-sized clusters, which might consist of several middle-sized agglomerates clumped together, as noted by Picard et al.

The SEM images also highlight the impact of the filler concentration on particle dispersion. While low-filler-content samples (PA6/OMMT-1 and PA6/OMMT-2.5 wt. %) show well-dispersed particles, higher-filler-content samples (PA6/OMMT-5 and PA6/OMMT-10 wt. %) display large, irregular agglomerates, consistent with predictions from the literature. This agglomeration effect may be responsible for the increased γ-phase formation observed in DSC analysis, where larger clusters promote heterogeneous nucleation, favoring the formation of γ-crystals. These results support the idea of the interphase model, where γ-crystalline phases could be coating the OMMT particles in an amorphous matrix, affecting the overall morphology. Earlier, the DSC results showed that the PA6/OMMT-5 and PA6/OMMT-10 wt. % nanocomposites could be dominated by γ-crystal forms. Based on this observation along with the nanostructure analysis, it can be assumed that the dominance of γ-crystal formations in the PA6/OMMT nanocomposites could be related to the formation of large clusters. Quantitative SEM image analysis was not performed in the present study due to the primary focus being on morphological trends and phase behavior as correlated with thermal properties.

### 3.4. Hydrogen Permeability of Nanocomposites

The hydrogen permeability tests were conducted using a constant-volume variable-pressure method at 2 MPa and 20 ± 1 °C. Although this test pressure is significantly lower than the operating pressure of commercial hydrogen storage vessels (up to 70 MPa), Sun et al. [10] demonstrated that polymeric hydrogen barrier materials can be reliably characterized under moderate pressure and temperature conditions, which are sufficient to capture the key trends in gas transport behavior without the need for high-pressure infrastructure.

Steady-state permeation was confirmed by observing a linear pressure increase on the permeate side over time. In all cases, data collection continued until a sufficiently linear region was obtained to extract the slope reliably for permeability calculation. A minimum testing time of 20 h was applied, depending on the sample’s permeability. For PA6/OMMT-10 wt. %, which showed the highest barrier performance, a 40 h duration was used to ensure that a sufficiently linear trend in the time–volume data could be established.

**Figure 7:**
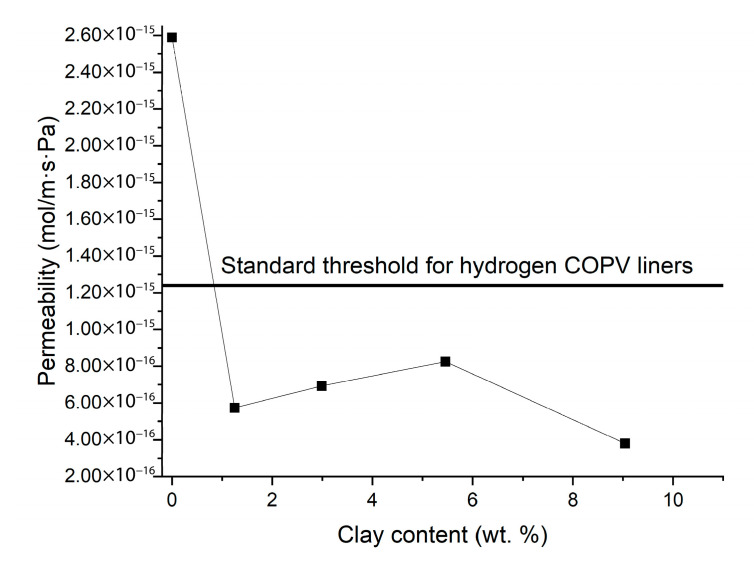
Hydrogen gas permeability coefficient of various PA6/OMMT composites.

The hydrogen permeability of neat PA6 was measured at 2.59 × 10^−15^ mol/m·s·Pa, a result which does not satisfy the ISO 19881-2018 standard of hydrogen containers. Sun et al. explains based on this standard that the hydrogen permeability coefficient must be below 1.24 × 10^−16^ mol/m·s·Pa [10]. As shown in Figure 7, the addition of OMMT significantly reduced the permeability across all compositions below the threshold. Among the 1, 2.5, and 5 wt. % OMMT-reinforced samples, the PA6/OMMT-1 wt. % composition exhibited the lowest permeability value (5.74 × 10^−16^ mol/m·s·Pa), suggesting that optimal clay dispersion plays a critical role in enhancing the gas barrier properties. This observation is consistent with the previously discussed morphological findings, where better exfoliation and distribution of OMMT were observed at lower filler contents.

Interestingly, although the PA6/OMMT-2.5 wt. % and PA6/OMMT-5 wt. % samples still exhibited lower permeability compared to neat PA6, their performance was weaker than that of the 1 wt. % sample, likely due to increased filler agglomeration and reduced crystallinity, as supported by the SEM and DSC results.

The PA6/OMMT-10 wt. % nanocomposite showed the lowest permeability overall (3.79 × 10^−16^ mol/m·s·Pa), indicating that a high filler content can create a highly tortuous diffusion path for hydrogen molecules, significantly hindering permeation. However, this improvement is primarily attributed to the physical barrier effect of densely packed fillers rather than uniform dispersion or high crystallinity.

The permeability results are in good agreement with previously reported values in the literature on PA6 and PA6 nanocomposites, as they all fall in the range of 10^−16^ to 10^−14^ mol/m·s·Pa depending on the test conditions and polymer grade [10,28].

These results highlight that while the incorporation of impermeable fillers inherently improves gas barrier properties, the quality of filler exfoliation and dispersion within the matrix remains crucial. Poor dispersion and filler aggregation can negate the positive impact on permeability reduction. Similar trends have been reported by Gupta et al. [29], who noted that the passive barrier effect is most effective when fillers are well exfoliated and homogeneously dispersed, whereas the presence of aggregates diminishes barrier performance. Furthermore, Gupta et al. demonstrated that permeability could be reduced by a factor of two with as little as 2 wt. % montmorillonite and by a factor of ten with 8 wt. % loading, closely aligning with the current findings.

Although the crystallinity generally decreased with increasing OMMT content, the permeability also decreased, which may appear contradictory at first glance. This phenomenon is explained by the dual contribution of organoclay: while an excessive filler load can hinder crystallization by disturbing polymer chain folding, it simultaneously forms a highly tortuous diffusion path and restricts chain mobility, which collectively dominate the transport behavior.

In low-filler-content systems (e.g., PA6/OMMT-1), both high crystallinity and exfoliated dispersion contribute to low permeability. In high-content systems (e.g., PA6/OMMT-10), despite the reduced crystallinity and severe agglomeration, the dense packing of clay particles elongates diffusion pathways and reduces the free volume for hydrogen molecules.

Therefore, permeability is governed not only by crystallinity but by the combined effects of the crystalline morphology, clay dispersion state, and polymer chain dynamics—all influenced by the OMMT content. This explains why different compositions achieve comparable or even superior barrier performance through distinct structural mechanisms.

## 4. Conclusions

This study examined the effects of varying organoclay content on the crystalline structure, morphology, and hydrogen permeability of injection-molded PA6/OMMT nanocomposites, focusing on their application in Type IV COPV liners. DSC analysis revealed that increased OMMT content and faster cooling rates promoted γ-phase formation, and lower organoclay concentrations favored α-phase crystallization. The crystallinity index calculations indicated a systematic decrease with higher organoclay content, except for the PA6/OMMT-1 wt. % sample, showing the highest crystallinity among all.

Peak deconvolution of the overlapped endothermic peaks confirmed a γ-phase increase with higher OMMT loading, particularly under rapid cooling. These thermal findings were supported by SEM imaging, which revealed γ-crystallites forming around clay platelets and a clear interphase structure. The correlation between WAXD and SEM was essential to assess the dispersion quality: exfoliation was confirmed for the PA6/OMMT-1 wt. % sample through both the disappearance of the low-angle diffraction peak and the presence of uniformly dispersed platelets in SEM images. In contrast, intermediate and high OMMT loadings showed restacking (WAXD) and agglomerates (SEM), indicating poorer dispersion. At 10 wt. %, disordered structures and micron-sized clusters were observed, highlighting the limits of filler loading.

Permeability analysis revealed that the addition of OMMT significantly reduced hydrogen permeability, particularly in the PA6/OMMT-1 wt. % and -10 wt. % samples. The PA6/OMMT-1 wt. % sample exhibited the most favorable combination of low permeability and high crystallinity, which is attributed to the optimal exfoliation and homogeneous dispersion of the clay platelets. In contrast, samples with intermediate filler loadings (2.5 and 5 wt. %) showed slightly higher permeability values, correlating with increased aggregation and reduced crystallinity.

These results reinforce the critical role of nanofiller dispersion quality and crystalline structure control in achieving superior gas barrier properties. Together, the thermal, morphological, structural, and permeability findings highlight the importance of controlling the OMMT content and cooling rates to tailor the crystalline structure and optimize the performance of PA6 nanocomposites in hydrogen storage applications.

## Figures and Tables

**Table 1:** Extrusion parameters.

Temperature Setting (°C)					Feeder Motor Speed (rpm)	Main Motor Speed (rpm)
Zone 1	Zone 2	Zone 3	Zone 4	Zone 5		
240	240	240	235	230	5	12

**Table 2:** Injection-molding parameters.

Nozzle Temperature (°C)	Mold Temperature (°C)	Injection Pressure (MPa)	Injection Time (s)	Dwell Time (s)
230	80	100	0.5	3

**Table 3:** Interlayer spacing (d_(001)_) of pristine OMMT and PA6/OMMT nanocomposites calculated from Bragg’s law.

Sample	2θ (°)	D-Spacing (Å)
Pristine OMMT	4.3	20.5
Neat PA6	-	No peak
PA6/OMMT-1 wt. %	-	No peak
PA6/OMMT-2.5 wt. %	3.3	26.8
PA6/OMMT-5 wt. %	2.5	35.3
PA6/OMMT-10 wt. %	-	Low-intensity peak

## Data Availability

The data presented in this study are available on request from the corresponding author.
